# Zinc-Enriched *Bifidobacterium longum* subsp. *longum* CCFM1195 Alleviates *Cutibacterium acnes*-Induced Skin Lesions in Mice by Mitigating Inflammatory Responses and Oxidative Stress

**DOI:** 10.3390/nu17111803

**Published:** 2025-05-26

**Authors:** Xiangyue Gu, Botao Wang, Tianmeng Zhang, Qiuxiang Zhang, Bingyong Mao, Xin Tang, Jianxin Zhao, Shumao Cui

**Affiliations:** 1 State Key Laboratory of Food Science and Resources, Jiangnan University, Wuxi 214122, China; 6220112022@stu.jiangnan.edu.cn (X.G.); zhangqx@jiangnan.edu.cn (Q.Z.); maobingyong@jiangnan.edu.cn (B.M.); xintang@jiangnan.edu.cn (X.T.); cuishumao@jiangnan.edu.cn (S.C.); 2 School of Food Science and Technology, Jiangnan University, Wuxi 214122, China; jnwangbotao@foxmail.com; 3 Bloomage Biotechnology Co., Ltd., Jinan 250101, China; zhangtm419@outlook.com; 4 International Joint Research Laboratory for Maternal-Infant Microbiota and Health, Jiangnan University, Wuxi 214122, China

**Keywords:** *Bifidobacterium longum* subsp. *longum* CCFM1195, acne vulgaris, zinc homeostasis, inflammation, oxidative stress

## Abstract

**Background:** Acne vulgaris, a prevalent inflammatory skin disorder, stems from factors like *Cutibacterium acnes* overgrowth, inflammation dysregulation, and immune dysfunction. Clinically, acne severity inversely correlates with serum zinc (Zn) levels, and oral Zn supplementation shows efficacy. Lactic acid bacteria are capable of converting inorganic Zn into organic forms via biological transformation, potentially generating Zn-enriched bacteria as superior Zn delivery vehicles. **Methods:** In this study, a Zn-deficient acne mouse model was established through dietary Zn restriction combined with intradermal *C. acnes* injection. The therapeutic effects of orally administered Zn-containing supplements, including Zn-enriched *Bifidobacterium longum* subsp. *longum* CCFM1195 (Zn-CCFM1195), were systematically evaluated through multiple parameters: histopathological evaluation of skin lesions, cutaneous inflammatory and oxidative stress markers, serum Zn concentration, and gene expression levels of pathway-associated proteins. **Results:** Induction of *C. acnes* led to decreased serum Zn levels (14.98 μmol/L in Control vs. 9.71 μmol/L in Model) and skin metallothionein content, causing Zn imbalance. Zn deficiency caused increased levels of lesion elevation (9.23 in Model vs. 10.53 in Zn-deficient Model), IL-17A, TNF-α, and MMP9 in skin, thereby exacerbating the inflammatory response in C. *acnes*-induced mice. Zn supplementation alleviated inflammatory responses and oxidative stress in Zn-deficient acne-like mice. Notably, inactivated Zn-CCFM1195 exhibited superior efficacy to ZnSO_4_, significantly reducing lesion diameter and decreasing cutaneous levels of IL-1β, IL-17A, and MDA while enhancing GSH-Px activity. Similarly, viable Zn-CCFM1195 treatment significantly decreased IL-17A and enhanced GSH-Px activity compared with ZnSO_4_ treatment. Furthermore, Zn supplementation downregulated the expression of TLR2, IκBα, and IKKβ, which may exert its anti-acne effect by regulating related pathways. **Conclusions:** Zn deficiency exacerbates skin inflammation, whereas Zn supplementation, particularly with Zn-CCFM1195, alleviates acne vulgaris through anti-inflammatory and antioxidant effects.

## 1. Introduction

Acne vulgaris, a widespread inflammatory skin disorder affecting approximately 90% of adolescents and 65% of adults, is characterized by polymorphic lesions including comedones, papules, pustules, and cysts [1,2]. Its pathogenesis is multifactorial, involving excessive inflammation triggered by *Cutibacterium acnes*, increased sebum production, oxidative stress, and follicular hyperkeratinization. *C. acnes* activates the TLR2 (Toll-like receptor-2) and NLRP3 (NOD-like receptor protein 3) inflammasome pathway to trigger proinflammatory cytokines, recruit neutrophils, and induce tissue damage. Concurrently, *C. acnes*-induced reactive oxygen species (ROS) overwhelm endogenous antioxidant defenses, creating a vicious cycle where oxidative stress amplifies inflammation and vice versa [3,4,5]. While current therapies targeting one or more of these steps show efficacy, long-term use often leads to antibiotic resistance or skin barrier compromise.

Zinc (Zn), an essential trace element, demonstrates multimodal therapeutic potential in acne management [6,7]. It directly inhibits *C. acnes* proliferation by disrupting bacterial membranes [8] and suppressing immune responses via NF-κB (nuclear factor kappa-B) signaling pathways modulation [9]. Zn deficiency exacerbates acne-related inflammation, as evidenced by elevated IL-1β levels in an acne-like mouse model [10]. Inflammation further disrupts Zn homeostasis, forming a pathological loop [11,12]. Clinical trials confirm that Zn supplementation reduces inflammatory lesions, with inorganic Zn (e.g., zinc sulfate, ZnSO_4_) requiring higher doses (>100 mg/day) than organic forms (like zinc gluconate) ranging from 30–100 mg/day [13,14,15,16]. However, high-dose Zn causes gastrointestinal toxicity and copper/iron deficiency [17]. Additionally, there is a clear need for high-efficacy, low-toxicity Zn supplementation strategies.

Zinc-enriched lactic acid bacteria (LAB) represent an innovative approach by combining Zn’s bioactivity with probiotic benefits. Oral administration of LAB [18] or Zn alone has shown efficacy in alleviating acne vulgaris. LAB convert inorganic Zn into bioavailable organic forms, reducing toxicity [19,20]. Zn-enriched *Lactobacillus* strains outperform inorganic Zn and non-enriched probiotics in antioxidant and anti-inflammatory activities [21,22]. However, the therapeutic potential of Zn-enriched LAB against acne remains unexplored.

Our previous work identified *Bifidobacterium longum* subsp. *longum* CCFM1195 as a highly efficient Zn accumulator [23]. We hypothesize that oral administration of Zn-enriched CCFM1195 (Zn-CCFM1195) alleviates acne synergistically. To test this, we established a Zn-deficient mouse acne-like model via Zn-deficient feeding and dorsal injection of *C. acnes*. The alleviating effect of Zn-CCFM1195 was evaluated alongside multiple controls: ZnSO_4_, non-Zn-enriched CCFM1195, and a physical mixture of CCFM1195+ZnSO_4_. Both viable and inactivated bacterial controls were included to ensure experimental reliability.

## 2. Materials and Methods

### 2.1. Bacterial Strains and Culture Conditions

*B. longum* subsp. *longum* CCFM1195 is a strain that is highly enriched with Zn, stored in the Culture Collection of Food Microorganisms (CCFM) of Jiangnan University [23]. The strain was inoculated into modified de Man, Rogosa, and Sharpe (MRS) medium containing 200 mg/L Zn^2+^ and incubated anaerobically at 37 °C for 16 h. Subsequently, the bacteria were harvested via centrifugation at 8000× *g* for 15 min at 4 °C, then rinsed with sterile ultrapure water to remove Zn^2+^ remaining on the cell surface, repeated twice. Finally, the bacteria were resuspended in 10% sucrose solution and freeze-dried to obtain Zn-enriched bacterial powder [23].

*C. acnes* ATCC6919, sourced from Guangdong Microbial Culture Center, was inoculated in Reinforced Clostridial Medium (RCM) and incubated at 37 °C for 48 h. The bacteria were subsequently centrifuged at 8000× *g* for 15 min at 4 °C and washed three times with PBS. The cells were finally resuspended in PBS to a concentration of 3.8 × 10^9^ CFU/mL for use in animal experiments [24].

### 2.2. Determination of Zn Content

The testing method for the Zn content of the Zn-enriched bacterial powder refers to the Chinese national standard GB 5009.268-2016 [25]. Zn-enriched samples were digested using a microwave digestion instrument (ETHOS UP, Milestone, Sorisole, Italy), then the digested solutions were diluted using ultrapure water. An inductively coupled plasma mass spectrometer (ICP-MS; NexION 350D, PerkinElmer, Waltham, MA, USA) was employed to determine the Zn content. The Zn standard material was purchased from Weiye Measurement Standard Material Research Center (Beijing, China).

The serum Zn content was determined using a fully automatic biochemical analyzer (BS-420, Shenzhen Mindray Biomedical Electronics Co., Ltd., Shenzhen, China).

### 2.3. Animal Experiment Design

Male Balb/c mice (*n* = 88, 7 weeks old, weighing 19–22 g) were sourced from Beijing Vital River Laboratory Animal Technology Co., Ltd. (Beijing, China). The mice were housed under controlled environmental conditions, maintaining a constant temperature of 20 °C ± 2 °C and relative humidity of 50% ± 5%, with a 12 h light–dark cycle. The experiment began after one week of adaptation. Normal feed (TP0690C, 38 mg Zn/kg) and low-Zn feed (TP0690-01, <1 mg Zn/kg) were produced by Jiangsu Nantong Trophy Animal Feed High-tech Co., Ltd. (Nantong, China) [26]. The research protocol was reviewed and approved by the Institutional Animal Care and Use Committee (IACUC) of the Jiangsu Provincial Institute of Parasitic Diseases (approval number: JIPD-IACUC-2024083). The sample size (*n* = 8 per group) was determined based on previous studies [27] and adheres to the ARRIVE guidelines for animal research.

All mice were randomly divided into 11 groups: (1) Control group (Control), (2) Model group (Model), (3) Zn-deficient Control group (Zn-de-Control), and (4) Zn-deficient Model group (Zn-de-Model). The remaining groups received Zn supplementation: (5) viable Zn-enriched CCFM1195 (vi-Zn-CCFM1195), (6) inactivated Zn-enriched CCFM1195 (in-Zn-CCFM1195), (7) viable CCFM1195 mixed with ZnSO_4_ (vi-CCFM1195+ZnSO_4_), (8) inactivated CCFM1195 mixed with ZnSO4 (in-CCFM1195+ZnSO_4_), and (9) Zn sulfate group (ZnSO_4_). Additionally, separate groups were established for bacterial treatments: (10) viable CCFM1195 group (vi-CCFM1195) and (11) inactivated CCFM1195 group (in-CCFM1195).

**Figure 1:**
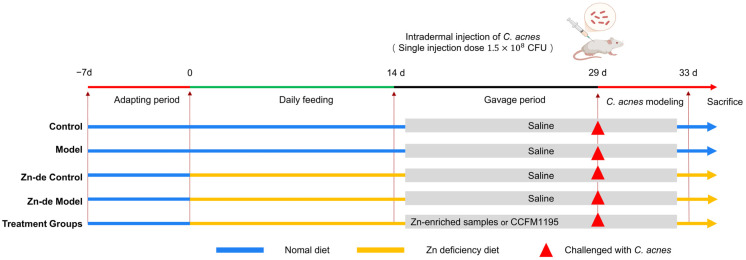
Design of the animal experiment.

The design of the animal experiments is shown in Figure 1. During the experiment, mice in the Control group and Model group were fed a normal diet, while the mice in the other groups were fed a low-Zn diet (≤1 ppm Zn). Based on the serum Zn content (see Figure S1), when the mice were in a state of Zn deficiency, from the 15th day to the end of the experiment, the Control group, the Model group, the Zn-de Control group, and the Zn-de Model group were given 0.2 mL normal saline orally. The remaining groups were orally supplemented with corresponding Zn-enriched samples or bacteria. The content of Zn in the normal diet was 38 ppm. According to the daily feed intake of mice (4 g), the Zn supplement in the Zn-enriched groups was 0.15 mg, and the number of viable bacteria in the viable bacteria groups was 1 × 10^9^ CFU/d. On the 29th day of modeling, the mice in the Control group and the Zn-de Control group were injected with normal saline in the back, and the other mice were intradermally injected with 40 μL (a single injection amount) of *C. acnes* (3.8 × 10^9^ CFU/mL) at 4 points in the back to induce acne-like lesions. The modeling method referred to the methods used in Rimon A [24] and Kolar S L [28]. On day 33, the mice were photographed (Huawei Nova 10, Huawei, Shenzhen, China, 50 MP) dorsally, anesthetized with isoflurane, subjected to orbital blood collection, and euthanized by cervical dislocation.

### 2.4. Assessment of the Severity of Acne Vulgaris

The resulting photographs were analyzed using ImageJ software (version 1.53t; National Institutes of Health, Bethesda, MD, USA) to determine the diameter of the acne lesions. Additionally, 10 individuals were enlisted to rate the swelling elevation of the lesions on a scale of 0 to 5, as shown in Figure S2 as an example. Histologic analysis of skin tissues was conducted using hematoxylin and eosin (H&E) staining. All images were visualized using the Pannormic MIDI (3DHistech Ltd., Budapest, Hungary) at 5× magnification.

### 2.5. Biochemical Analysis of Skin Tissue

After the mice were killed, 2 cm^2^ sections of the skin were cut along the edge of the wound for tissue acquisition. The skin tissue samples were mixed with PBS at a ratio of 1:9 (*w*/*v*). The tissue samples were then homogenized using a high-throughput tissue grinder at a frequency of 65 Hz for 30 s per cycle, totaling 8 cycles. Subsequently, the homogenates were centrifuged at 1016× *g* for 15 min at 4 °C to obtain the supernatant for subsequent determination. Assays for tumor necrosis factor-α (TNF-α), interleukin-1β (IL-1β), interleukin-17A (IL-17A), C-X-C motif chemokine ligand 1 (CXCL1/KC), matrix metalloproteinase-9 (MMP9), and metallothionein (MT) content were conducted using commercial ELISA [29] kits supplied by Mlbio (Shanghai, China) or Multisciences (Hangzhou, China). Malondialdehyde (MDA) was determined based on a thiobarbituric acid reactive substances (TBARS) assay (Solebol kit, Beijing Solarbio Science and Technology Co., Ltd., Beijing, China) [30]. Copper/Zn superoxide dismutase (Cu/Zn SOD), SOD, and glutathione peroxidase (GSH-Px) were analyzed using WST-8 and DTNB methods, respectively (Beyotime kits, Beyotime Institute of Biotechnology, Shanghai, China). The BCA method was used for protein normalization (Beyotime).

### 2.6. Quantitative qPCR Analysis

The total RNA of skin tissues was extracted using the trizol method [31]. Subsequently, a commercial reverse transcription kit (Vazyme, Nanjing, China) was used to convert total RNA into complementary DNA (cDNA). The ChamQ Universal SYBR qPCR Master Mix (Vazyme, China) was employed to configure the fluorescence quantitative PCR reaction system (10 µL), composed of 5 µL of SYBR qPCR Master Mix, 0.25 µL of upstream primers, 0.25 µL of downstream primers, 3.5 µL of ddH_2_O, and 1 µL of DNA template. The RT-qPCR reaction program was as follows: 95 °C for 30 s, 40 cycles (95 °C for 30 s; 60 °C for 30 s), and a dissolution curve from 65 °C to 95 °C, incremented by 0.5 °C/s. *GAPDH* was used as the internal reference gene, and the target gene expression level was determined based on the 2^−∆∆Ct^. The primers applied in this study are detailed in Table 1.

### 2.7. Statistical Analysis

GraphPad Prism 9 (GraphPad Software, LLC, San Diego, CA, USA) was used for statistical analysis and graph preparation. When the data had a normal distribution, one-way analysis of variance (ANOVA) was utilized to compare the differences between groups of more than two. Fisher’s LSD test was employed to evaluate statistically significant variations across groups. When the data were not normally distributed, the Brown–Forsythe test was used to compare the medians between the groups [31]. *p* < 0.05 was considered significant. The data are presented as means ± standard deviations (SDs).

## 3. Results

### 3.1. Viable Bacteria Count and Zn Content of CCFM1195 Powder

The Zn content of the freeze-dried powder of Zn-CCFM1195 was 8.80 mg/g, and its viable count was 9.1 × 10^10^ CFU/g. The number of viable bacteria in the freeze-dried powder of CCFM1195 was 2.9 × 10^10^ CFU/g.

### 3.2. Zn-Enriched Samples Ameliorate Skin Injury in Mice

**Figure 2:**
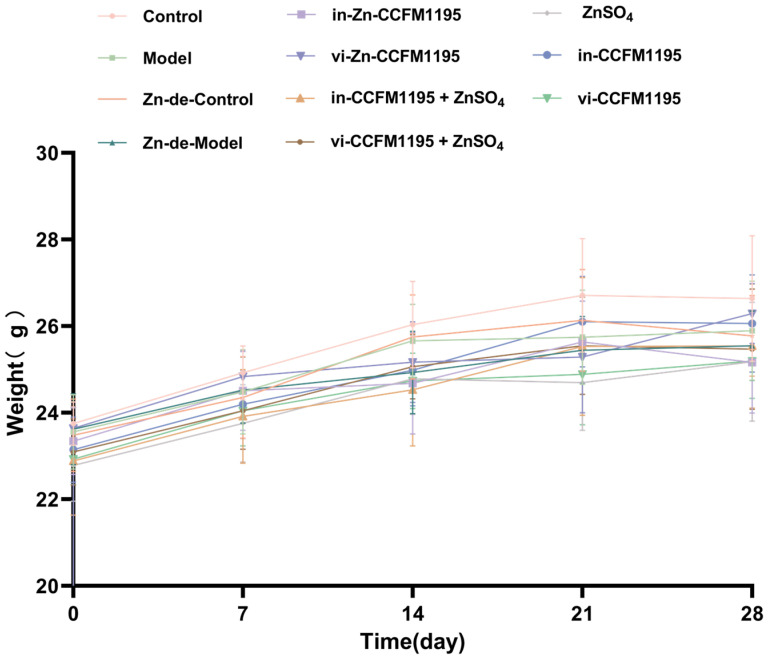
Body weight before *C. acnes* treatment.

**Figure 3:**
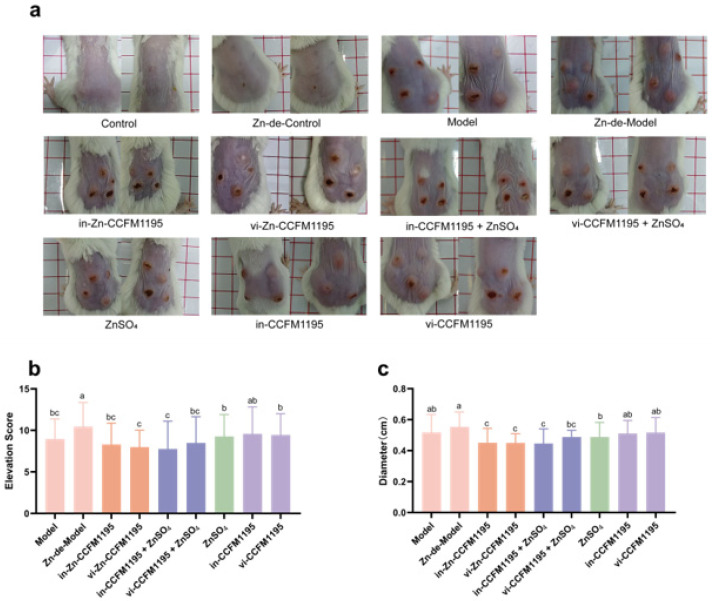
Characterization of *C. acnes*-induced lesions in mice. (**a**) Dorsal images of mice before sacrifice, (**b**) lesion elevation scores, (**c**) lesion diameters. The results are presented as means ± SDs. Different superscript letters indicate statistically significant differences among groups (*p* < 0.05).

We evaluated the severity of the lesions by measuring the diameter and calculating elevation scores. Figure 2 indicates there was no significant difference in body weight between groups during the Zn deficiency and Zn supplementation phases. After intradermal injection of *C. acnes*, the Control and Zn-de-Control groups exhibited no significant skin injury, whereas the Model and Zn-de-Model groups developed pustules on the dorsal skin, with the Zn-de-Model group showing greater severity (Figure 3a). Compared with the Zn-de-Model group, the Zn supplement groups demonstrated reduced pustule size, increased scarring, and significantly lower elevation scores and diameters (Figure 3). The Zn-CCFM1195 (including in-Zn-CCFM1195 and vi-Zn-CCFM1195) and CCFM1195+ZnSO_4_ (including in-CCFM1195+ZnSO4 and vi-CCFM1195+ZnSO4) showed a more pronounced reduction in elevation scores compared to ZnSO_4_ alone, though no significant differences were observed among these four groups (Figure 3b). The lesion diameters in the groups treated with in-Zn-CCFM1195, vi-Zn-CCFM1195, in-CCFM1195+ZnSO_4_, and vi-CCFM1195+ZnSO_4_ were significantly reduced to 0.45, 0.44, 0.45, and 0.49 cm, respectively, compared to the 0.55 cm observed in the Zn-de-Model group (Figure 3c). Meanwhile, both in-CCFM1195 and vi-CCFM1195 interventions resulted in a slight reduction in lesions elevation scores and diameter in mice, though their effects were less pronounced compared to Zn-CCFM1195 and CCFM1195+ZnSO_4_.

### 3.3. Zn-Enriched Samples Affect Skin Histopathology

**Figure 4:**
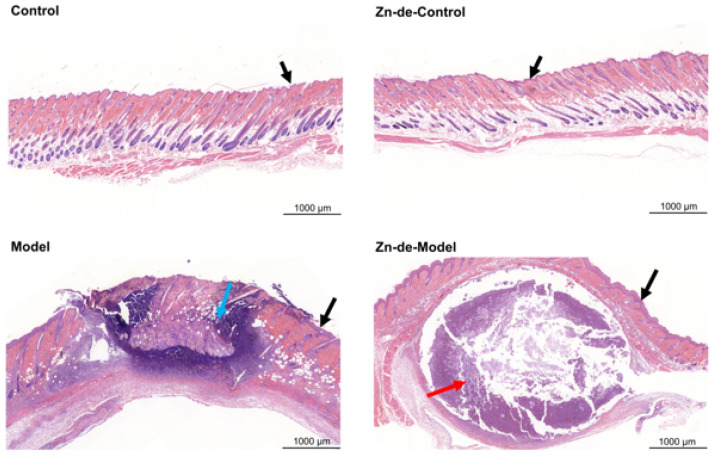
Histopathological changes after injection of *C. acnes* (scale bar, 1000 μm and magnification, 5×). Blue arrows indicate dermal inflammatory cell infiltration, red arrows indicate subcutaneous inflammatory cell infiltration, and black arrows indicate the epidermis.

**Figure 5:**
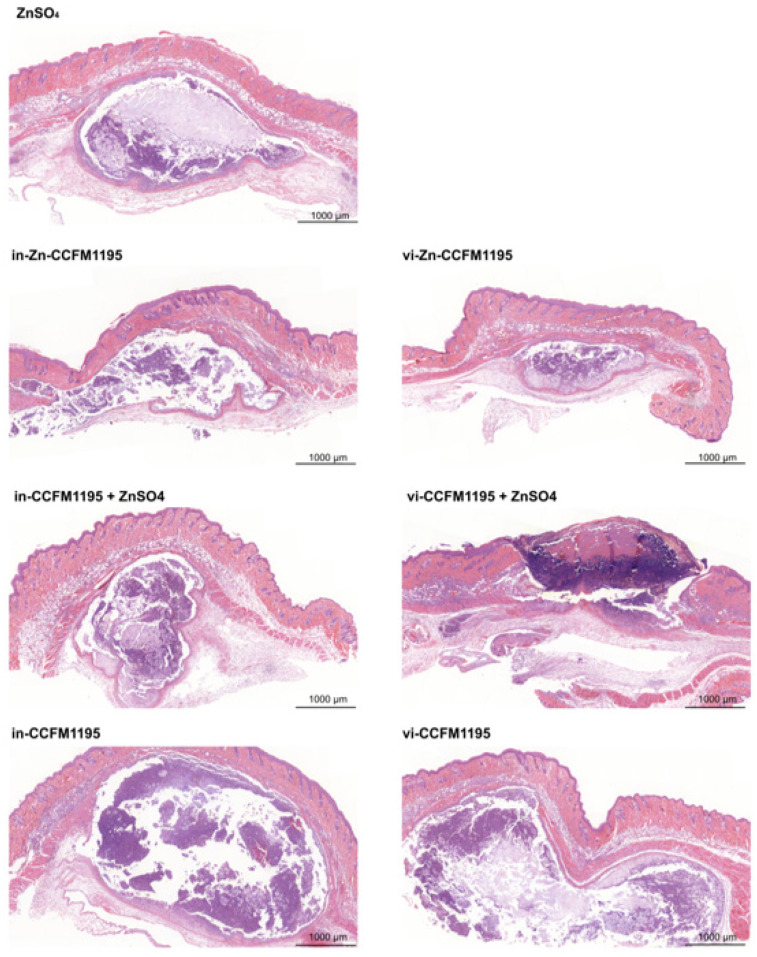
Histopathological changes after sample intervention (scale bar, 1000 μm; magnification, 5×).

As shown in Figure 4, in the sham-injected groups, the epidermis appeared intact with uniform thickness, and no significant abnormalities were observed in the dermis or subcutaneous tissue. In contrast, *C. acnes*-induced modeling resulted in acute inflammation, characterized by an abundance of granulocytes and necrotic cell debris in the dermis (indicated by blue arrows) or subcutaneous area (indicated by red arrows), forming localized abscesses. In the Model and Zn-de-Model groups, pronounced epidermal thickening was observed near the injection site, which was significantly greater than that in both the Control and Zn-de-Control groups (indicated by black arrows). As shown in Figure 5, compared with the Model and Zn-de-Model groups, Zn supplementation significantly reduced inflammatory cell infiltration. However, neither in-CCFM1195 nor vi-CCFM1195 treatment showed significant effects compared to the Zn-de-Model. Importantly, the Zn-CCFM1195 and CCFM1195+ZnSO_4_ groups demonstrated more restricted inflammatory infiltration than ZnSO_4_ alone. Epidermal thickness remained unchanged across all treatment groups.

### 3.4. Analysis of Zn Status Indicators in Serum and Skin After Zn Supplementation

**Figure 6:**
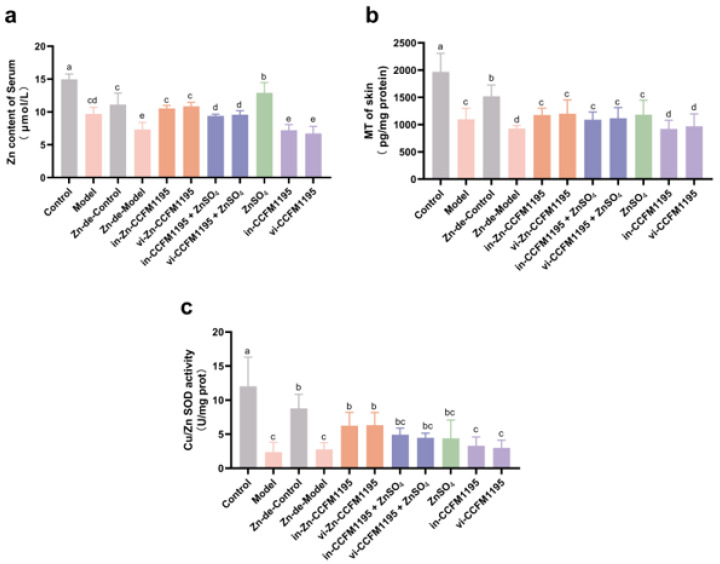
Zn status indicators of serum and skin. (**a**) Zn content in serum, (**b**) MT content in skin, (**c**) Cu-Zn SOD activity in skin. The results are presented as means ± SDs. Different superscript letters indicate statistically significant differences among groups (*p* < 0.05).

Subsequently, we focused on the Zn status indicators in the serum and skin. Induction by *C. acnes* led to decreased serum Zn levels (14.98 μmol/L in Control vs. 9.71 μmol/L in Model) and skin MTcontent (1970.25 pg/mg prot in Control vs. 1099.63 pg/mg prot in Model), causing Zn imbalance. Dietary Zn deficiency and *C. acnes* infection synergistically exacerbated Zn depletion, with the Zn-de-Model group showing 34.18% lower serum Zn (*p* = 0.0016) and 38.86% reduced skin MT (*p* < 0.0001) compared to Zn-de-Controls (Figure 6a,b). Compared to the Zn-de-Model group, the ZnSO_4_ group demonstrated the most significant restoration of serum Zn levels, followed by Zn-CCFM1195, then CCFM1195+ZnSO_4_ (Figure 6a). Figure 6b demonstrates that skin MT levels were significantly elevated in the ZnSO_4_, Zn-CCFM1195, and CCFM1195+ZnSO_4_ groups compared to the Zn-de-Model group, although no significant intergroup differences were observed among these treatment groups. The activity of Cu/Zn SOD, a Zn-dependent enzyme, decreased after *C. acnes* modeling and tended to recover following Zn supplementation. Among them, Zn-CCFM1195 had a significant improvement compared to the Zn-de-Model group (Figure 6c).

### 3.5. Zn-Enriched Samples Ameliorate Inflammatory Response of Skin

**Figure 7:**
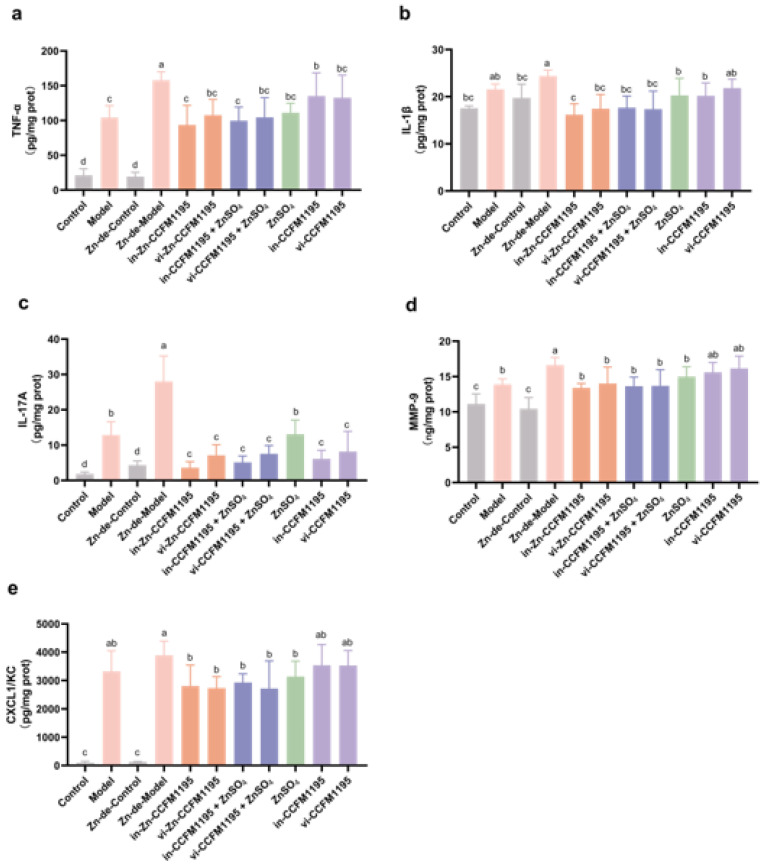
Effects of Zn products on inflammatory mediators in the skin. (**a**) TNF-α levels, (**b**) IL-1β levels, (**c**) IL-17A levels, (**d**) MMP9 levels, (**e**) CXCL1/KC levels. The results are presented as means ± SDs. Different superscript letters indicate statistically significant differences among groups (*p* < 0.05).

As shown in Figure 7, after *C. acnes* modeling, the levels of pro-inflammatory cytokines (TNF-α, IL-1β, and IL-17A), MMP9, and chemokine CXCL1/KC were significantly elevated in the skin of mice compared to controls. The Zn-de-Model group exhibited higher levels of these cytokines than the Model group, with increases of 51.74% for TNF-α, 11.91% for IL-1β, 118.06% for IL-17A, 19.64% for MMP9, and 17.05% for CXCL1/KC, indicating that Zn deficiency leads to more severe skin inflammation in acne-like mice. ZnSO_4_ treatment led to a significant reduction in these factors, but Zn-CCFM1195 and CCFM1195+ZnSO_4_ were more effective, with in-Zn-CCFM1195 outperforming ZnSO_4_ in TNF-α (*p* = 0.0137), IL-1β (*p* = 0.0127), and IL-17A (*p* = 0.0060). Both viable and inactivated CCFM1195 treatments reduced TNF-α and IL-1β to levels comparable to ZnSO_4_ and also significantly decreased IL-17A, to a lesser extent than ZnSO_4_.

To further assess the effects of Zn supplementation on mice modeled with *C. acnes*, we quantified the mRNA levels of genes related to inflammation. As shown in Table 2, Zn deficiency significantly exacerbated the expression of calcium-binding proteins (*S100A8* and *S100A9*), *TLR2*, *MyD88*, *IκBα*, and *IKKβ* in acne-like mice, and Zn supplementation attenuated these effects. Among the Zn treatments, ZnSO_4_ demonstrated the least efficacy, while other Zn-containing supplements exhibited comparable effects. Notably, compared with ZnSO_4_, in-Zn-CCFM1195 treatment reduced the mRNA expression of *S100A8*, *S100A9*, *TLR2*, and *IκBα* by 35.17%, 50.34%, 66.22%, and 47.42%, respectively. Similarly, vi-Zn-CCFM1195 treatment decreased the mRNA expression of *S100A8*, *TLR2*, and *IκBα* by 50.99%, 32.37%, and 60.65%, respectively, compared with ZnSO_4_ treatment. Additionally, CCFM1195 alone also significantly reduced the expression of certain genes, including *TLR2*, *IκBα*.

### 3.6. Zn-Enriched Samples Improve Oxidative Stress in Skin

In comparison to the Zn-de-Control group, the MDA levels in the Zn-de-Model group were significantly increased by 79.36%; GSH-Px and SOD activities were significantly decreased by 37.97% and 67.68%, respectively (Table 2). Compared to Zn-de-Model group, supplementation with ZnSO_4_ resulted in elevations in GSH-Px activity and SOD activity and a moderate decrease in MDA levels. However, compared to ZnSO_4_,the Zn-CCFM1195 and CCFM1195+ZnSO_4_ treatments exhibited greater improvements in these oxidative stress markers, particularly for GSH-Px activity. Notably, in-Zn-CCFM1195 demonstrated a 27.36% (*p* = 0.0372) greater reduction in MDA levels and a 47.64% (*p* = 0.0026) greater increase in GSH-Px activity relative to ZnSO_4_ treatment. Meanwhile, vi-Zn-CCFM1195 demonstrated a 34.80% (*p* = 0.0154) greater increase in GSH-Px activity relative to ZnSO_4_ treatment. Concerning antioxidant genes, the expression of *Nrf2* was diminished post-modeling but was upregulated following Zn supplementation. Zn-CCFM1195 most markedly elevated the expression of *Nrf2*, followed by CCFM1195+ZnSO_4_, with ZnSO_4_ showing the least improvement.

## 4. Discussion

Zn is recognized for its role in alleviating acne vulgaris [7]. However, most research has focused on conditions with relatively high Zn levels, and comparative studies on different Zn forms are rare. In this study, we assessed the efficacy of various Zn-containing supplements in a dietary Zn restriction combined with intradermal *C. acnes* injection induced mouse model of acne vulgaris. Our findings show that *C. acnes* induction decreases serum Zn levels in mice, consistent with the reduced levels observed in clinical studies with acne patients (78.9 μg/dL in patients vs. 105.5 μg/dL in healthy controls) [17,29]. This suggests that risk factors for acne vulgaris may lead to Zn imbalances in the body. In addition, mice in the Zn-de-Model displayed more severe skin lesion than controls (Figure 3 and Figure 4), supporting the findings of Li et al. [10], who found a significant dose–response relationship between dietary Zn intake and disease severity. In our study, Zn-CCFM1195 and CCFM1195+ZnSO_4_ were superior to ZnSO_4_ in alleviating skin lesions and reducing inflammatory infiltration of neutrophils (Figure 3 and Figure 4).

*C. acnes* is one of the key causative factors of acne vulgaris and is thought to stimulate immune responses in the skin. Post-*C. acnes* induction, the mice in the different groups exhibited varying degrees of skin injury characterized by redness, swelling, crusting, and inflammatory cell infiltration. Patients with clinical pustular acne are categorized into three stages: Stage 1 features large, soft pustules; Stage 2 shows a reduction in pustule size and content, with scab formation; Stage 3 is marked by scab resolution [32]. Thus, scab formation may indicate that wounds induced by the modeling are undergoing repair. Zn is involved in all phases of wound healing and facilitates the healing process [33]. Xiang et al. [34] demonstrated that a nanoparticle ZnO patch stimulated the release of Zn ions in fibroblasts, up-regulated DNA replication-related genes, enhanced fibroblast proliferation, and consequently accelerated skin repair in acne-like mice. This explains the increased skin scab formation observed in the Model and Zn-supplemented mice compared to the Zn-de Model mice (Figure 3a). By evaluating swelling (elevation and diameter) and inflammatory cell invasion, we determined that Zn-CCFM1195 and CCFM1195+ZnSO_4_ outperformed ZnSO_4_ in alleviating acne (Figure 3 and Figure 4).

Zn morphology and other components of the diet influence Zn absorption and tissue distribution. In previous studies, Zn-CCFM1195 and CCFM1195+ZnO yielded higher serum and tissue Zn levels than ZnO alone [23]. However, this study found that serum Zn levels were highest for ZnSO_4_, followed by Zn-CCFM1195, and then CCFM1195+ZnSO_4_ (Figure 6a), potentially due to the selection of different inorganic Zn sources. Fortified cereals demonstrated greater Zn absorption from ZnSO_4_ than ZnO, attributed to the higher bioaccessibility of ZnSO_4_ [35]. Alternatively, the slow-release effects of Zn-CCFM1195 and CCFM1195+ZnO on absorption, confirmed in prior studies [23], may play a role, as bacterial macromolecules like proteins and carbohydrates must be metabolized to release Zn for gradual absorption [36]. The serum Zn outcomes in this experiment might reflect that Zn absorption by Zn-CCFM1195 and CCFM1195+ZnSO_4_ had not peaked at the time of sampling. MT, a Zn-binding protein with broad tissue expression [37], exhibits variable expression in response to dietary Zn levels [38]. It has been shown that MT levels are more sensitive to response in mild Zn-deficient diets than tissue Zn levels [39]. Cu/Zn SOD, a Zn-containing enzyme, serves as a biomarker for Zn deficiency and supplementation, reflecting its activity in serum [40] and tissues [39]. Consequently, in the skin, we assayed MT content and Cu/Zn SOD activity as Zn markers. The MT levels and Cu/Zn SOD activity of Zn-CCFM1195 and CCFM1195+ZnSO_4_ were similar to those observed with ZnSO_4_ (Figure 6b,c). Thus, variations in the absorption of different Zn supplement forms may not affect skin Zn homeostasis in this model. Nonetheless, it is clear that *C. acnes* modeling reduced serum and skin Zn marker levels and activities, which increased following Zn supplementation.

The beneficial effects of Zn on acne vulgaris may be related to the anti-inflammatory and antioxidant activities of Zn [7]. TNF-α and IL-1β, well-established pro-inflammatory cytokines, are produced early in the inflammatory response and are crucial throughout the process [41]. Their levels were significantly decreased in the skin of acne-like mice following supplementation with Zn-containing samples (Figure 7a,b). Research indicates that the IL17/Th17 pathway is activated in acne lesions [42], with IL-17A being highly expressed in the epidermis and lymphocytes of patients with acne vulgaris [40]. *C. acnes* stimulates the expression of Th17-related genes, including IL-17A [43]. Zn has been shown to alter T-cell differentiation and regulate Th17/Treg balance, thereby regulating IL-17 [44,45]. In colitis, Zn deficiency exacerbates colonic inflammation by activating Th17 cells in mice [46]. In our study, both Zn deficiency and *C. acnes* induced a decrease in skin IL-17A levels (Figure 7c), potentially a key factor in acne vulgaris exacerbation due to Zn deficiency. Supplementation with CCFM1195 and ZnSO_4_ alone reduced IL-17A in the skin, and in-Zn-CCFM1195 showed a synergistic effect of the CCFM1195 and Zn (Figure 7c). MMPs act on pro-inflammatory cytokines, chemokines, and other proteins, facilitating movement of inflammatory cells toward damaged sites [47]. The catalytic domains of MMPs contain Zn ions, and both Zn deficiency and excess modulate MMP activity and expression [48]. Chemokines specifically attract and recruit immune effector cells to the site of injury or infection, thereby triggering an inflammatory response [49]. Our study found that Zn-CCFM1195 and CCFM1195+ZnSO_4_ more effectively reduced MMP9 and CXCL1/KC than ZnSO_4_ (Figure 7d,e). In addition, *S100A8* and *S100A9* expression were significantly upregulated in the model induced by *C. acnes* [50]. They are released during cellular stress or tissue injury and act as damage-associated molecular patterns (DAMPs) or alarmins [51]. In our study, Zn significantly reduced the gene expression of *S100A8* and *S100A9* following treatment with Zn-CCFM1195 and CCFM1195+ZnSO_4_ (Table 2). The TLR2/NF-κB pathway is highly expressed in acne vulgaris [52]. The typical NF-κB complex consists of a p65-p50 heterodimer and IκBα. Phosphorylation of IκBα by the IKK complex leads to its ubiquitination and proteasomal degradation, activating the NF-κB dimer, which then translocates to the nucleus and upregulates target gene expression [9]. Zn can exert anti-inflammatory effects by directly inhibiting IKK and inducing cGMP, thereby inhibiting the NF-κB signaling pathway [53]. Accordingly, the Zn-containing samples in this study significantly reduced the expression of *TLR2*, *IκBα*, and *IKKβ*, with greater efficacy than ZnSO_4_ (Table 2). Thus, Zn-containing samples may attenuate the inflammatory response in acne-like mice by inhibiting the TLR2/NF-κB pathway.

There is crosstalk between oxidative stress and inflammation, with studies demonstrating oxidative damage in the serum markers of acne vulgaris patients [54]. Nrf2, a key transcription factor, regulates the expression of a variety of antioxidant genes through antioxidant response elements (AREs) [55]. Zn-containing samples significantly increased the expression of *Nrf2* and showed that Zn-CCFM1195 was superior to CCFM1195+ZnSO_4_ (Table 2). This upregulation may correspondingly have led to increased GSH-Px activity in Zn-containing samples. MT, suggested to have antioxidant effects due to its Cys-rich and Zn^2+^-binding via sulfhydryls [56], can serve as a Zn marker. The increase in MT levels after Zn supplementation may also contribute to enhanced antioxidant capacity in acne-like mice (Figure 6b). In conclusion, amelioration of the inflammatory response and oxidative stress may be responsible for the improvement of acne vulgaris in Zn-containing samples.

Our findings indicate that Zn-CCFM1195 could serve as a novel Zn supplement for acne vulgaris patients with Zn deficiency. While the acne-like mouse model showed promising efficacy, translational human applications require further investigation, particularly regarding gut–skin axis regulation differences, optimal zinc dosing to avoid overexposure, and gastrointestinal side effects, and there are differences between human and mouse skin physiology, especially in pilosebaceous unit density and immune responses. Future clinical trials should assess long-term safety and efficacy to bridge these gaps.

## 5. Conclusions

In this study, we investigated the impact of *C. acnes* on Zn homeostasis and compared the efficacy of Zn-CCFM1195, CCFM1195+ZnSO_4_, and ZnSO_4_ in alleviating acne vulgaris. Our results demonstrated that *C. acnes* modeling disrupts Zn homeostasis in mice, with Zn deficiency intensifying acne symptoms and inflammation. Zn-CCFM1195 and CCFM1195+ZnSO_4_ alleviated acne by modulating the inflammation and redox status of mouse skin, which were superior to ZnSO_4_. These are expected to become new Zn supplements for Zn-deficient acne patients.

## Figures and Tables

**Table 1:** The primers used in the experiment.

zGene	Primer Sequence (5′-3′)
*GAPDH*	Forward: 5′-GGCAAATTCAACGGCACAGTCAAG-3′ Reverse: 5′-TCGCTCCTGGAAGATGGTGATGG-3′
*S100A8*	Forward: 5′-TGCCGTCTGAACTGGAGAAGG-3′ Reverse: 5′-CTTGTAGAGGGCATGGTGATTTCC-3′
*S100A9*	Forward: 5′-TGACATCATGGAGGACCTGGACAC-3′ Reverse: 5′-TGGGTTGTTCTCATGCAGCTTCTC-3′
*TLR2*	Forward: 5′-CTCCCAGATGCTTCGTTGTTCCC-3′ Reverse: 5′- GTTGTCGCCTGCTTCCAGAGTC-3′
*MyD88*	Forward: 5′-AGCAGAACCAGGAGTCCGAGAAG-3′ Reverse: 5′-GGGCAGTAGCAGATAAAGGCATCG-3′
*IκBα*	Forward: 5′-CTGAAAGCTGGCTGTGATCCTGAG-3′ Reverse: 5′-CTGCGTCAAGACTGCTACACTGG-3′
*IKKβ*	Forward: 5′-ATAAATTGCTGCTGGCTTGG-3′ Reverse: 5′-AGTGCCATCATCCGCTCTAC-3′
*GPX2*	Forward: 5′-AGCCTCAAGTATGTCCGACCTG-3′ Reverse: 5′-GGATGCTCGTTCTGCCCATTG-3′
*Nrf2*	Forward: 5′-CCTCACCTCTGCTGCAAGTA-3′ Reverse: 5′-TCAAATCCATGTCCTGCTGGG-3′

**Table 2:** The results and significance of antioxidative abilities and relative mRNA expression of genes related to inflammation and antioxidation.

	Group	Control	Model	Zn-de-Control	Zn-de-Model	in-Zn-CCFM1195	vi-Zn-CCFM1195	in-CCFM1195 + ZnSO_4_	vi-CCFM1195 + ZnSO4	ZnSO_4_	in-CCFM1195	vi-CCFM1195
Parameter												
*S100A8*		1.12 ± 0.73 ^e^	159.17 ± 54.92 ^d^	1.31 ± 0.41 ^e^	547.61 ± 91.54 ^a^	196.12 ± 49.64 ^d^	148.27 ± 38.73 ^d^	141.22 ± 34.13 ^d^	134.83 ± 9.32 ^d^	302.49 ± 77.77 ^c^	434.55 ± 31.49 ^b^	469.05 ± 52.17 ^ab^
*S100A9*		0.82 ± 0.25 ^e^	72.05 ± 12.23 ^d^	1.17 ± 0.44 ^e^	308.01 ± 87.48 ^a^	132.49 ± 6.40 ^c^	179.22 ± 44.25 ^bc^	142.74 ± 24.99 ^c^	129.16 ± 31.33 ^c^	266.77 ± 25.35 ^ab^	217.77 ± 15.43 ^b^	222.28 ± 63.08 ^b^
*TLR2*		1.10 ± 0.48 ^e^	2.51 ± 0.30 ^c^	2.27 ± 0.20 ^c^	4.45 ± 0.32 ^a^	0.96 ± 0.15 ^e^	1.92 ± 0.35 ^d^	2.01 ± 0.58 ^cd^	1.98 ± 0.20 ^d^	2.84 ± 0.96 ^c^	3.06 ± 0.69 ^bc^	3.75 ± 0.42 ^b^
*MyD88*		1.34 ± 0.70 ^c^	5.50 ± 1.33 ^b^	2.75 ± 0.46 ^c^	8.95 ± 2.01 ^a^	5.76 ± 1.46 ^b^	6.43 ± 1.90 ^b^	7.08 ± 2.13 ^ab^	8.52 ± 2.51 ^ab^	6.93 ± 1.10 ^b^	8.09 ± 1.36 ^ab^	8.85 ± 1.65 ^a^
*IκBα*		1.02 ± 0.12 ^d^	12.76 ± 3.43 ^c^	1.89 ± 0.69 ^d^	31.82 ± 3.33 ^a^	10.51 ± 1.95 ^c^	7.87 ± 3.46 ^c^	13.41 ± 4.22^c^	12.10 ± 5.48 ^c^	20.00 ± 3.13 ^b^	19.59 ± 6.75 ^b^	23.04 ± 2.26 ^b^
*IKKβ*		1.22 ± 0.55 ^c^	4.00 ± 0.95 ^b^	1.61 ± 0.52 ^c^	6.75 ± 1.94 ^a^	2.30 ± 1.67 ^bc^	2.28 ± 0.91 ^bc^	2.69 ± 1.36 ^bc^	2.95 ± 1.02 ^bc^	3.38 ± 1.65 ^b c^	5.18 ± 1.25 ^ab^	4.37 ± 1.65 ^b^
MDA (nmol/mg prot)		0.37 ± 0.1 ^c^	0.81 ± 0.09 ^a^	0.45 ± 0.08 ^bc^	0.81 ± 0.13 ^a^	0.49 ± 0.03 ^c^	0.60 ± 0.10 ^bc^	0.59 ± 0.14 ^bc^	0.56 ± 0.08 ^b^	0.67 ± 0.02 ^a b^	0.68 ± 0.16 ^ab^	0.77 ± 0.04 ^a^
GSH-Px activity (U/mg prot)		40.44 ± 3.82 ^ab^	29.54 ± 5.14 ^b^	40.85 ± 7.25 ^a^	23.16 ± 1.93 ^b^	46.48 ± 3.21 ^a^	42.44 ± 6.07 ^a^	39.94 ± 6.80 ^ab^	43.77 ± 9.27 ^a^	31.48 ± 1.48 ^b^	25.61 ± 3.95 ^b^	27.56 ± 1.75 ^b^
SOD activity (U/mg prot)		22.68 ± 6.50 ^a^	9.60 ± 3.67 ^bc^	21.87 ± 1.50 ^a^	7.07 ± 1.38 ^c^	12.35 ± 1.32 ^b^	11.87 ± 1.82 ^b^	10.80 ± 1.45 ^bc^	12.42 ± 3.51 ^b^	10.29 ± 2.49 ^bc^	9.43 ± 1.90 ^bc^	10.91 ± 1.37 ^bc^
*Nrf2*		1.05 ± 0.20 ^bc^	0.33 ± 0.22 ^d^	1.24 ± 0.12 ^ab^	0.61 ± 0.08 ^c^	1.27 ± 0.16 ^ab^	1.43 ± 0.18 ^a^	1.03 ± 0.22 ^bc^	1.10 ± 0.22 ^b^	0.80 ± 0.13 ^c^	0.60 ± 0.05 ^c d^	0.62 ± 0.09 ^cd^

All data are presented as means ± SDs. “^a–e^”: different letters indicate significant differences (*p* < 0.05) among different groups.

## Data Availability

The data used to support the findings of this study are available from the corresponding author upon request. The data are not publicly available as they include proprietary research data covered by confidentiality agreements with collaborating institutions.

## Supplementary Materials

The following supporting information can be downloaded at: https://www.mdpi.com/article/10.3390/nu17111803/s1, Figure S1: Serum zinc levels after 14 days of a Zn-deficient diet; Figure S2: Scoring criteria for lesion elevation.
