# Supplementary material for: Zinc-Enriched Bifidobacterium longum subsp. longum CCFM1195 Alleviates Cutibacterium acnes-Induced Skin Lesions in Mice by Mitigating Inflammatory Responses and Oxidative Stress
Source: Nutrients. 2025 May 26;17(11):1803. doi: 10.3390/nu17111803 (PMC12157163; doi:10.3390/nu17111803)
Supplement: Supplementary file 1 [file nutrients-17-01803-s001.zip › nutrients-3580815-supplementary.pdf]

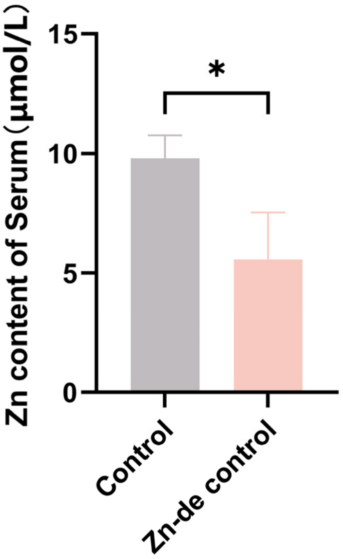

Figure S1. Serum Zn content after 14 days of a Zn-deficient diet. "*" indicates that there are significant differences between the two groups ($p < 0.05$).

Serum Zn levels were significantly lower after 14 days of a Zn-deficient diet, so subsequent Zn supplementation.

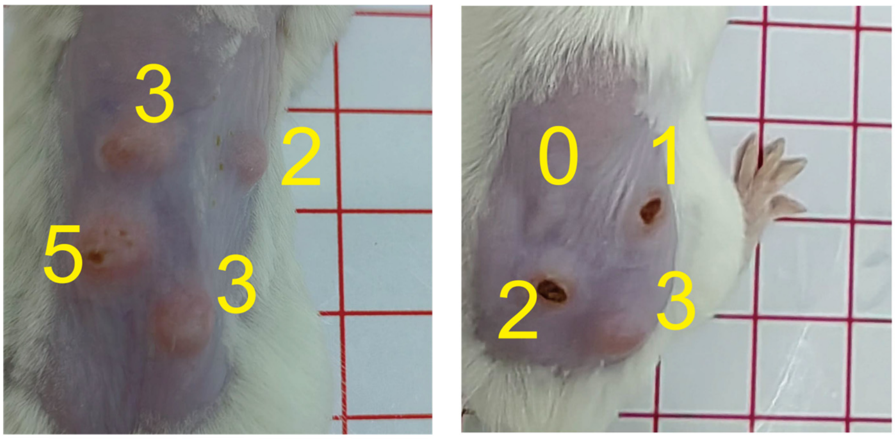

Figure S2. Scoring criteria for lesion elevation.
